# Location of Mental Foramen in Dentate Adults using Orthopantomogram

**DOI:** 10.31729/jnma.3692

**Published:** 2018-08-31

**Authors:** Barsha Ghimire, Sujaya Gupta

**Affiliations:** 1 Department of Prosthodontics, Kantipur Dental College, Basundhara, Kathmandu, Nepal; 2 Department of Periodontics, Kantipur Dental College, Basundhara, Kathmandu, Nepal

**Keywords:** *local anaesthesia*, *mental foramen*, *orthopantomogram*, *panoramic radiograph*

## Abstract

**Introduction:**

Understanding the anatomical variations in the position of mental foramen are significant for different dental procedures. This study identified the position of the mental foramen among a sample of Nepalese population visiting a dental college in Kathmandu.

**Methods:**

Total 417 panoramic radiographs (orthopantomograms) were selected from a total of 567 radiographs. The mental foramen location was determined by drawing imaginary line parallel with the long axis of the lower premolars. The mental foramen location was then classified into six classes.

**Results:**

In the study population, the mental foramina were located mostly between the lower premolars 163 (39.1%), followed by in line with second premolar 148 (35.5%) of the mental foramen was located under the second premolar apex.

**Conclusions:**

The study shows that the anaesthetic solution should be injected between the lower premolars or below the lower 2nd premolar in the Nepalese population for successful and secure mental nerve blocking.

## INTRODUCTION

The mental foramen (MF) is a strategically important landmark during dental procedures. Its location and the possibility that an anterior loop of the mental nerve may be present mesial to the mental foramen needs to be considered before implant surgery to avoid mental nerve injury.^1^

The accurate identification of the mental foramen is important for both diagnostic and clinical procedures. The radiographic appearance of the mental foramen may result in a misdiagnosis of a radiolucent lesion in the apical area of mandibular premolar teeth. Clinically, the mental bundle could be injured during surgical procedures, resulting in paraesthesia or anaesthesia. Generally the mental foramen is difficult to locate.^2^ In addition, knowing the exact location of mental foramen will help in achieving better local anaesthesia and pain control during dental treatment.

As the location of the mental foramen in Nepalese population has not been described previously, this study was undertaken to determine the most common position of the mental foramen in a selected population using orthopantomograms (OPGs). Therefore, the goal of our study was to identify the anatomical variations of mental foramen positions via panoramic radiographs in patients attending Kantipur dental college (KDC), Basundhara, Kathmandu.

## METHODS

This cross-sectional study was conducted using the records from Kantipur dental college, Basundhara, Kathmandu, Nepal. This study was reviewed and approved by the Institutional Research Committee (IRC) board at KDC.

A total of 567 OPGs from year 2017 to 2018 were retrieved from the medical record section and was evaluated by examiner. Sample size was calculated using the following formula:^3^


$$\text{SS = }\frac{{\text{Z}}^{2}\;\times \text{ P (1}-\text{ P)}}{{\text{e}}^{2}}$$

Z = 1.96 at confidence level 95%, P = 0.559^4^, e = Margin of error 0.05%

Patients aged from 20–70 years were considered in the study. Of these, 417 met the inclusion criteria. These criteria included: dentulous area, presence of two premolars and a first molar without periapical radiolucency on both sides, mental foramen was clearly visible at least on one side and a clear radiograph in terms of density and opacity. If one of these criteria was missing, then the radiograph was rejected. Exclusion criteria included severe crowding or spacing in lower arch, presence of radiolucent lesions in lower jaws, fracture lines involving parasymphyseal region. All radiographs were assessed on x-ray view box by the principal investigator to ensure the consistency. The mental foramen location was determined by drawing imaginary lines parallel with the long axis of the lower premolars and mesial root of the first molar.^4,5^ Relative to the drawn line, the mental foramen position was documented.

The foramen was usually larger on the left side of the mandible. Based on its radiographic appearance, the mental foramen has been classified by Yosue and Brooks^6^ into four types:

Type I: mental canal is continuous with the mandibular canal
Type II: the foramen is distinctly separated from the mandibular canal
Type III: diffuse with a distinct border of the foramen Type IV: “unidentified group”

The position of the image of the mental foramen was recorded as by Ngeow and Yuzawati into six positions:^2^

Position 1: Situated anterior to the first premolar
Position 2: In line with first premolar
Position 3: Between the first and second premolar
Position 4: In line with second premolar
Position 5: Between the second premolar and first molar
Position 6: In line with first molar

The statistical analyses were performed using Statistical Package for Social Sciences (SPSS) software program for Windows version 20.0 Armonk, NY:IBM Corp. SPSS Statistics. Qualitative data are presented as frequencies and percentages and quantitative data as means and standard deviations. The level of significance was set at 0.05.

## RESULTS

Of the 567 panoramic radiographs analysed, 417 showed a mental foramen. In the remaining, the mental foramen was not clearly visible. These radiographs were excluded from the study.

The median age of the patients was 28 years with age range from 20 years to 70 years. In our study 61% were female (255) with female to male ratio of 1.56. The mean size of mental foramen was 4.05± 0.85 ranged from 2mm to 7mm.

**Table 1:** Sex distribution among different age group.

Age Categories	Female n (%)	Male n (%)	Total	Mean±S.D.	S.E. Mean
20 to 35	212 (50.8)	132 (31.7)	344 (82.5)	30.06±8.773	0.430
36 and above	42 (10.1)	31 (7.4)	73 (17.5)		
Total	254 (60.9)	163 (39.1)	417		

**Table 2:** Size of mental foramen in relation to sex.

	Mean	Standard Deviation	Standard Error Mean	P
Female	3.97	0.814	0.051	0.06
Male	4.13	0.897	0.070	

Independent t-test (2-tailed) at confidence interval 95%.

**Table 3:** Sex and Mental foramen relation to Mandibular canal.

MC Relation	Female n (%)	Male n (%)	Total	P
Continuous	85 (20.4)	57 (13.7)	142 (34.1)	0.349
Diffuse	4 (1)	6 (1.4)	10 (2.4)	
Separated	165 (39.6)	100 (24)	265 (63.5)	
Total	254 (60.9)	163 (39.1)	417	

A separated appearance was most common observation 265 (63.4%) in our study, followed by continuous 142 (34.1%) and diffuse 10 (2.4%).

The most common position for the mental foramen relative to the teeth was between first and second premolar 163 (39.1%). The second most common location was in line with second premolar 148 (35.5%). The third between first molar and second premolar 58 (13.9%), fourth in line with first premolar 45 (10.8%), fifth was in line with second premolar 3 (1.2%) and sixth was in line with first molar 3 (0.7%).

When position of mental foramen was compared between male and female the most common position was found between the lower premolars.

**Figure 1.**
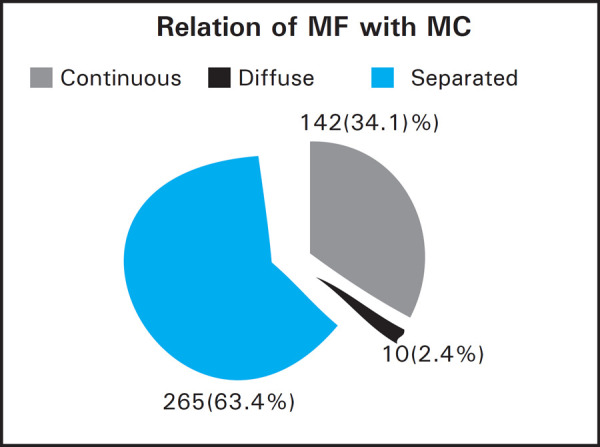
Relationship of Mental foramen with Mandibular canal.

**Figure 2.**
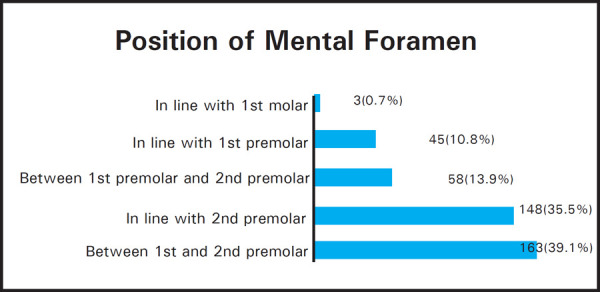
Position of mental foramen.

**Table 4:** Mental foramen position among different sexes.

Mental Foramen Position	Female n (%)	Male n (%)	Total n (%)	P value
Between 1^st^ and 2^nd^ premolar	103 (24.7)	60 (14.4)	163 (39.1)	P = 0.026
In line with 2^nd^ premolar	96 (23)	52 (12.5)	148 (35.5)	
Between 1^st^ molar and 2^nd^ premolar	27 (6.5)	31 (7.4)	58 (13.9)	
In line with 1^st^ premolar	28 (6.7)	17 (4.1)	45 (10.8)	
In line with 1^st^ molar	0	3 (0.7)	3 (0.7)	
Total	254 (60.9)	163 (39.1)	417 (100)	

## DISCUSSION

The present study provides new data on the position of the mental foramen in the Nepalese population. Anatomically, the mental foramen is the opening of the short mental canal, a branch of the mandibular canal (MC). Although on most standardized panoramic radiographs, the radiographic landmarks of the mental foramen can be seen, the appearance of these landmarks varies without any change of radiographic quality.

Panoramic radiographs were utilized because they have certain advantages over intra-oral radiography.

These include a greater area of hard and soft tissue and also the ability to visualize adjacent areas, thus allowing for a more accurate localization of the mental foramen in both the horizontal and vertical dimensions. Periapical radiographs do not reveal the position of the mental foramen if it falls below the edge of the film. Our study was limited to adult patients, because in a mixed dentition, permanent tooth buds might obscure the mental foramen.^7^

This study demonstrates that the mental foramen can easily be identified on panoramic radiographs. However, as the bone density increases, the foramen becomes more difficult to identify and it may not be seen clearly even with optimal illumination. Yosue and Brook classified these examples as the 'unidentified type.^6^ Ngeow has found out the most common position for mental foramen was in line with the longitudinal axis of the second premolar followed by a location between the first and second premolar.^2^ Currie et al determined the most common position for the mental foramen between the first and second premolar teeth.^8^ Similarly Shah also revealed most common position of mental foramen is between first and second premolars.^9^ Suragimath et al did a similar study and concluded that the separate type of mental foramen was most predominant and the most common location was position 4 followed by position

When panoramic radiographs were taken with proper patient position, there usually will be limited horizontal overlap of teeth. However, variations in facial characteristics of patients, associated with growth and development as well as errors in patient positioning, can lead to mesial or distal angulation of the X-ray beam.^5^ The distortion and magnification factors inherent in the orthopantomogram techniques cannot be eliminated if the image is too sharp. Since inter patient variation with respect to position in the focal plane will always be present, the distortion and magnification will consequently vary from one patient to another. The radiation beam of the panoramic machine comes from the lingual side of the mandible.^11^ Therefore, there would be a greater separation between the apex and the mental foramen because of the buccal object rule.^12^

Determining the positional variation of mental foramen is important for isolation of mental nerves and vessels when administering local anaesthesia and performing surgeries.^13^ The most frequent appearance of MF in our study was separated type which showed variation from previous reports. The results of our study supports only few previous reported studies concerning the most frequent horizontal and vertical locations of mental foramen, which clearly indicates that it has positional variations in different population groups. These findings can be used as reference material by the dental practitioners of Nepal while performing clinical procedures that involve mental nerves and vessels.

## CONCLUSIONS

The study shows that the anaesthetic solution should be injected between the lower premolars or below the lower 2nd premolar in the Nepalese population for successful and secure mental nerve blocking.
